# Giant cell tumor of tendon sheath at the hand: A case report and literature review

**DOI:** 10.1016/j.amsu.2020.09.011

**Published:** 2020-09-09

**Authors:** Zhengming Lv, Jie Liu

**Affiliations:** Department of Orthopaedics, Taizhou People's Hospital, 366 TaiHu Road, Taizhou, 225300, Jiangsu, China

**Keywords:** Giant cell tumor of tendon sheath, Surgical excision, Pulley, Palmaris longus tendon

## Abstract

**Introduction:**

GCTTS is the second most popular soft tissue tumor at the hand next to ganglion cyst, and also named tenosynovial giant cell tumor or pigmented villonodular tenosynovitis. It is divided into localized form and diffuse form. We introduce a report of a rare case of GCTTS in a female where lesions were identiied within the left ring finger and also conducted a literature review.

**Presentation of case:**

We describe a 32-year-old female patient with GCTTS a single digit since six months. Radiographic and histopathological examination is necessary to help determine whether to take further treatment. Surgical excision was performed, including complete removal of the tumor and reconstruction of the pulley with autologous tendon. Histopathology suggested that these masses were consistent with GCTTS without malignancy. There was no clinical and radiologic evidence of recurrence six months after surgery.

**Discussion:**

GCTTS is a benign fibrous tissue tumor originating from the tenosynosheath, bursae and joint synovium. This tumor is more common in adults aged 30–50, and is slanted toward females. The major risk of GCTTS is recurrence and joint damage, which requires surgical resection. The integrity of the pulley plays an important role in the function of the hand. In this case, the ipsilateral metacarpal tendon was taken during the operation to reconstruct the pulley to reduce the possibility of loss of hand function.

**Conclusion:**

This case represents a rare case of GCTTS at the hand within a single digit. Due to its high recurrence rate, the tumor should be completely removed to reduce the possibility of recurrence. Radiographic and histopathological examination must be performed on the tumor, which is determined to be benign and does not require further treatment. The function of the hand should be reconstructed to minimize the loss if necessary.

## Highlights

• We describe a 32-year-old female patient with GCTTS a single digit since six months.
• Preoperative examinations including clinical examination and imaging examination were improved.
• The scope and size of the tumor were fully evaluated to determine the surgical method.
• We completely excised the masses and the involved tendon sheath.
• The part of the ipsilateral palmaris longus tendon was used to reconstruct the A2 pulley.
• Postoperative pathology was performed to determine the nature of the tumor.
• The tumor should be completely removed to reduce the possibility of recurrence.

## 1 Introduction

**Fig. 1:**
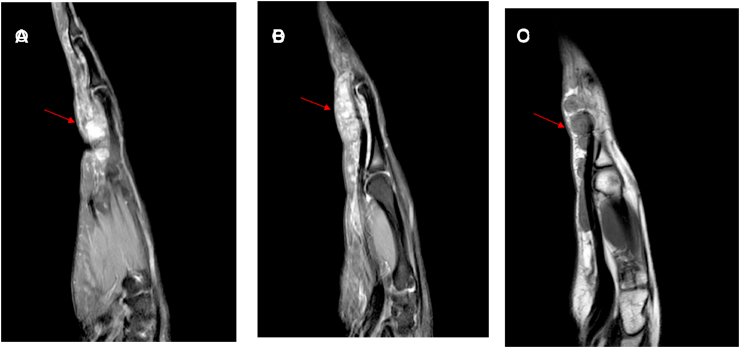
(A,B,C) MRI showed these masses on the surface of the flexor tendens.

GCTTS is the second most popular soft tissue tumor at the hand next to ganglion cyst, and also named tenosynovial giant cell tumor (TSGCT) or pigmented villonodular tenosynovitis (PVNS) [1,2]. According to clinical and biological manifestations, it is divided into localized form and diffuse form. Also there are two types: intra-articular and extra-articular. Localized form is benign and involve the hand and fingers, while diffuse form is more aggressive and occurs in large joints [3]. GCTTS typically occurs near the distal interphalangeal joint of the index or long finger of the hand [4]. (see Fig. 1)

Here, we introduce a report of a rare case of GCTTS in a female where lesions were identiied within the left ring finger and also conducted a literature review. This report was approved by the hospital ethics committee and conducted with the informed consent of patient and her families. The work has been reported in line with the SCARE criteria [5].

## 2 Presentation of case

A 32-year-old female, right hand dominant, visited to our clinic with a six-month history of left ring finger discomfort and obvious growing masses. Based on physical examination, the nodules were 7 × 0.7 × 0.5 cm in size at the volar aspect of the left ring finger and the fourth metacarpal. Physical examination showed mild pain and swelling; These masses were irregular in shape and unclear from the surrounding tissue; however, no paresthesia was noted, and normal circulation of the capillaries were present. The motion of the proximal interphalangeal and metacarpophalangeal joint were moderately restricted, and the motion of the distal interphalangeal joint is normal. The smooth glide and grip strength of left hand were worse than right hand. The patient has no history of smoking and alcohol abuse, and no family member has any history of related diseases.

X-ray examination showed the mass shadow without bone erosion. Magnetic resonance imaging (MRI) showed the size and extent of these masses. These masses ranged from the middle part of the fourth metacarpal bone to the proximal middle phalanx of left ring finger. Preoperative tumor indicators were normal.

**Fig. 2:**
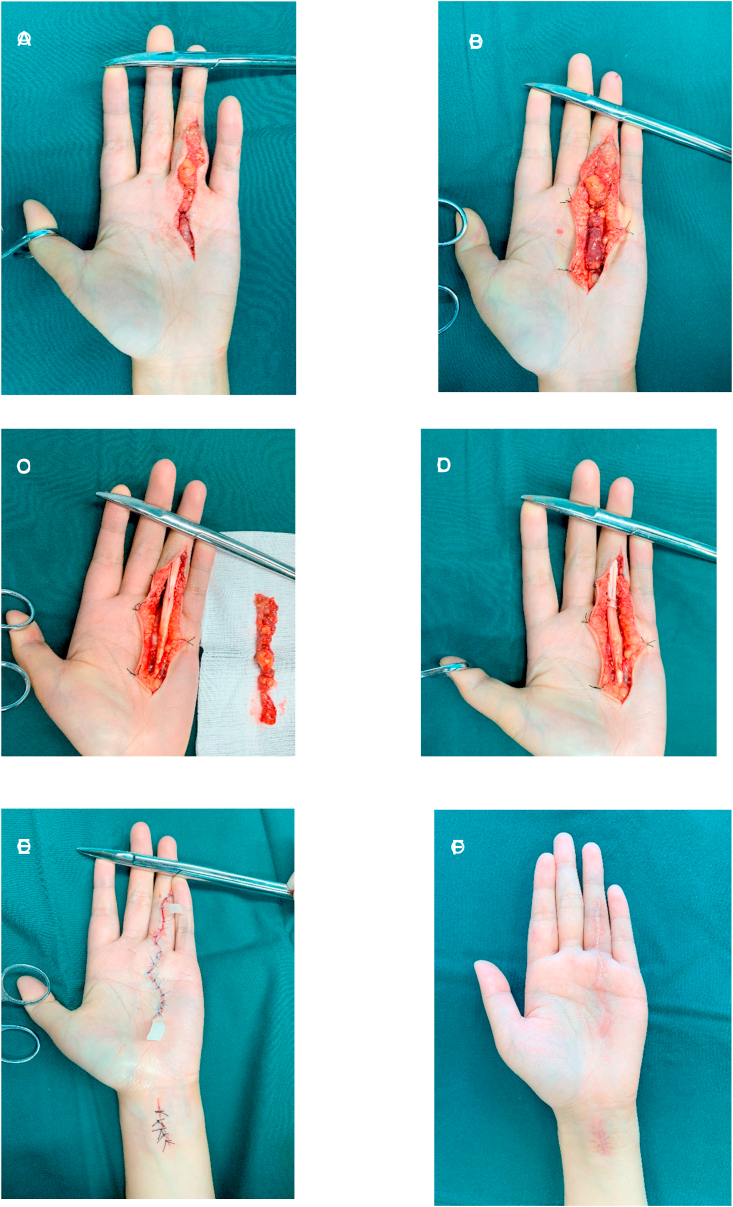
(A)Incised along the tumor surface. (B)Clearly revealed the masses at the volar aspect of the left ring finger and the fourth metacarpal. (C)Completely excised the masses and the involved tendon sheath. (D)Cut out the ipsilateral palmaris longus tendon to reconstruct the A2 pulley. (E)Closed the wound and placed the flap drainage. (F)Hand photos were followed up six months after surgery.

According to the patient's requirements and symptoms, surgical treatment was attempted by two orthopaedic attending physicians, both of whom have more than 3 years of working experience. Surgical treatment was attempted. We performed a Z-shaped incision on the surface of these masses. The masses at the volar aspect of the left ring finger and the fourth metacarpal were clearly revealed. These masses closely surrounded the flexor tendons. The tendon sheath and bony were slightly corroded by the masses. We completely excised the masses and the involved tendon sheath. The part of the ipsilateral palmaris longus tendon was used to reconstruct the A2 pulley. The wound healed completely without infection after the operation. One week after surgery, she complied with doctor's advice to start rehabilitation exercise to reduce adhesion of tendon tissues. Postoperative rehabilitation exercise included passive activity three days after surgery and active activity one week after surgery (see Fig. 2).

**Fig. 3:**
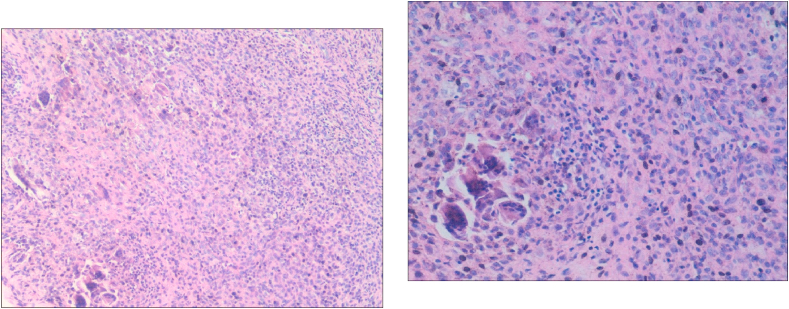
Microscopic examination of the tumor showed mononuclear cells and inflammatory cells in a hyalinized collagenous matrix, with multiple scattered multinucleated giant cells (hematoxylin and eosin stain; 100 × magnification [left], 200 × magnification [right]).

Histopathologic examination showed that the tumor was lobulated and surrounded by dense and transparent collagen. The intratumoral cells were composed of a mixture of histiocytoid monocytes, osteoblastic multinucleate giant cells, xanthoma cells, chronic inflammatory cells, ferruginous phagocytes and collagenous matrix (Fig. 3). Histopathology suggested that these masses were consistent with GCTTS without malignancy.

Postoperative follow-up was conducted every three months, the patient expressed her gratitude that there was no deformity or numbness that might occur in preoperative communication. The patient made a full recovery without significant swelling and restricted movement. The flexion and extension of the metacarpophalangeal joint and proximal interphalangeal joint of the ring finger were normal without bowstring deformity six months after the operation. The smooth glide and grip strength of left side were similar to right side. Meanwhile, there was no clinical and radiologic evidence of recurrence.

## 3 Discussion

GCTTS is a benign fibrous tissue tumor originating from the tenosynosheath, bursae and joint synovium [2]. This tumor is more common in adults aged 30–50, and is slanted toward females [6]. The etiology of GCTTS is uncertain, which may be related to inflammatory reaction process, local lipid metabolism disorder, osteoclastic proliferation, trauma, infection, ect [7]. GCTTS presents painless, swelling,slow-growing masses in general.

The major risk of GCTTS is recurrence and joint damage, which requires surgical resection [8]. Choughri et al. considered that the recurrence rate of tumor after surgery was about 15–45% [9]. Williams et al. reported the overall recurrence rates ranged from 7 to 44% [10]. Hakan recorded the recurrences were 6% [11]. At present, radical surgical management is particularly important to reduce recurrence. In our study these lesions with tendon sheaths has been achieved complete excision to prevent recurrence. No recurrence was found at the six-month follow-up, which was continued in the following study. Here we highlight that the tumor with tendon sheath and bone erosions should be removed radically.

Manske and Tang JB et al. suggested hand function would be partially lost because of the removal of A1 and A2 pulley [12,13]. Therefore, the integrity of the pulley plays an important role in the function of the hand. In this case, the ipsilateral metacarpal tendon was taken during the operation to reconstruct the pulley to reduce the possibility of loss of hand function. In hand tumors, the surgeon did not only remove the lesion, but also should consider the reduction of functional loss by reconstruction. Postoperative observation of the patient's hand flexion and extension activities were normal, no bowstring deformity occurred.

## 4 Conclusion

Our case represents a rare case of GCTTS at the hand within a single digit. Moreover, because of its high recurrence rate, the tumor should be completely removed to reduce the possibility of recurrence. In addition, radiographic and histopathological examination must be performed on the tumor, which is determined to be benign and does not require further treatment. Finally, the function of the hand should be reconstructed to minimize the loss if necessary.

## Ethical approval

This report was conducted in compliance with ethical standards. Informed written consent has been obtained and all identifying information is omitted.

## Sources of funding

None.

## Author contributions

Zhengming Lv-Operation of case, conception of study, drafting of manuscript Jie Liu-Modification of manuscript, approval of the final version for submission.

## Registration of research studies

This is a case report study.

## Guarantor

Huimin Liang (lianghuimin889757@163.com).

## Provenance and peer review

Not commissioned, external peer review.

## Informed consent

Informed written consent has been obtained and all identifying information is omitted.

## Declaration of competing interest

No conflicts of interests.

## Funding

This research did not receive any specific grant from funding agencies in the public, commercial, or not-for-profit sectors.
